# Epidemiology of *Strongyloides stercoralis* in northern Italy: results of a multicentre case–control study, February 2013 to July 2014

**DOI:** 10.2807/1560-7917.ES.2016.21.31.30310

**Published:** 2016-08-04

**Authors:** Dora Buonfrate, Mara Baldissera, Fabrizio Abrescia, Matteo Bassetti, Giacomo Caramaschi, Mario Giobbia, Marta Mascarello, Paola Rodari, Novella Scattolo, Giuseppina Napoletano, Zeno Bisoffi, on behalf of the CCM Strongyloides Study Group

**Affiliations:** 1 Centre for Tropical Diseases, Sacro Cuore Hospital, Negrar (Verona), Italy; 2 Prevention Department, ULSS20, Verona, Italy; 3 Medici per la Pace Onlus, Verona, Italy; 4 University Hospital of Udine, Udine, Italy; 5 Servizio di Medicina di Laboratorio, Azienda Ospedaliera Carlo Poma, Mantova, Italy; 6 Infectious Diseases Department, Ca’ Foncello Hospital, Treviso, Italy; 7 Department of Infectious Diseases, University Hospital of Trieste, Trieste, Italy; 8 University Department of Infectious and Tropical Diseases, University of Brescia and Spedali Civili General Hospital, Brescia, Italy; 9 Laboratorio Analisi, Ospedale Fracastoro, San Bonifacio (Verona), Italy; 10 Members of the group are listed at the end of the article

**Keywords:** Epidemiology, parasitic disease, *Strongyloides stercoralis*, Italy

## Abstract

*Strongyloides stercoralis* is a soil-transmitted helminth widely diffused in tropical and subtropical regions of the world. Autochthonous cases have been also diagnosed sporadically in areas of temperate climate. We aimed at defining the epidemiology of strongyloidiasis in immigrants and Italians living in three northern Italian Regions. Screening for *S. stercoralis* infection was done with serology, confirmation tests were a second serological method or stool agar culture. A case–control approach was adopted and patients with a peripheral eosinophil count ≥ 500/mcL were classified as cases. Of 2,701 individuals enrolled here 1,351 were cases and 1,350 controls; 86% were Italians, 48% women. Italians testing positive were in 8% (97/1,137) cases and 1% (13/1,178) controls (adjusted odds ratio (aOR) 8.2; 95% confidence interval (CI): 4.5–14.8), while positive immigrants were in 17% (36/214) cases and in 2% (3/172) controls (aOR 9.6; 95% CI: 2.9–32.4). Factors associated with a higher risk of infection for all study participants were eosinophilia (p < 0.001) and immigration (p = 0.001). Overall, strongyloidiasis was nine-times more frequent in individuals with eosinophilia than in those with normal eosinophil count.

## Introduction

*Strongyloides stercoralis* is a soil-transmitted helminth affecting millions of people worldwide [1,2]. Its transmission occurs in areas where poor hygienic conditions and humid, warm climate permit the free-living cycle of the parasite. The larvae present in the soil can penetrate human skin, therefore barefoot walking and agricultural activities pose people at risk of acquiring the infection. *S. stercoralis* produces larvae that can reinfect the host by a so called auto-infective cycle, a peculiarity shared only by *Capillaria* spp [3], so that an infected person remains infected life-long, if not properly treated [4]. This is the reason why strongyloidiasis can be diagnosed in people who have left endemic countries already several years before.

The few studies conducted in the United States (US) and in Europe to evaluate the prevalence of strongyloidiasis in immigrants and refugees from endemic countries, either through population or hospital-based studies, probably underestimated the real burden of the infection as long as microscopic stool examination was the only test used for screening [5]. In fact, the methods commonly employed for stool microscopy such as formalin-ether concentration, have a low sensitivity. Preferred faecal-based methods for the detection of *S. stercoralis* are Baermann funnel concentration and agar plate culture (APC), but the method that has so far demonstrated the highest sensitivity is serology [6]. Studies conducted in the field, classically underestimate the burden of strongyloidiasis if there is no special focus on this infection i.e. through using an appropriate diagnostic method. This is why the ‘old’ estimates of prevalence from the late 1980s and 1990s [7,8] were recently questioned [1,2].

The transmission of strongyloidiasis occurs especially in tropical and subtropical areas. However, in some temperate countries, autochthonous transmission occurred in the past [9,10], or might be still ongoing [11,12]. Therefore, cases of *S. stercoralis* infection can be diagnosed in people who have never moved from the Mediterranean coast.

Strongyloidiasis can be fatal in immunocompromised patients so prompt diagnosis and effective treatment are crucial for all those infected, in order to prevent later complications, such as disseminated strongyloidiasis [4]. Chronic infection is characterised by mild, unspecific symptoms such as pruritus, abdominal pain or discomfort, respiratory impairment which are not easily attributable to *S. stercoralis* and there is no full agreement among experts on considering eosinophilia as a predictor of the infection [13]. However, in non-endemic countries a high eosinophil count might be a sufficient index of suspicion in travellers or in patients over 65 years with history of barefoot walking in a formerly endemic area [14,15].

The treatment of choice for strongyloidiasis is ivermectin that has demonstrated a higher efficacy than albendazole [16]. Although the drug is included in the World Health Organization (WHO) list of essential medicines [17], it is not accessible for the vast majority of infected people in the world [18,19]. In fact, this essential drug is still donated to endemic countries, but with the strict limitation of use for *Wuchereria bancrofti* and *Onchocerca volvulus* control programmes [20]. In Italy, ivermectin has never been registered for human use.

In a previous pilot study, we screened 132 Italian individuals born in 1940 or earlier, with eosinophilia and no significant travel history, presenting to the clinical laboratories of two health districts. The serology test, an in-house immunofluorescence antibody test (IFAT), was positive in 28% of cases, suggesting that strongyloidiasis can be a relevant cause of eosinophilia in this group of individuals [9].

In the present study, we extended the previous screening in order to estimate the prevalence of strongyloidiasis in six provinces of three Italian Regions. The population analysed included both adult immigrants and Italians born before 1952, with or without eosinophilia.

## Methods

### Study design and setting

We conducted a multicentre case–control study.

**Figure 1:**
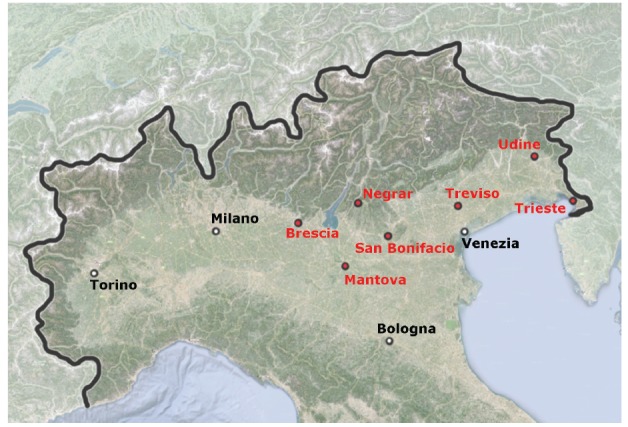
Map of northern Italy showing where participating sites are situated, study of *Strongyloides stercoralis* epidemiology, northern Italy, February 2013–July 2014

Participants were enrolled between 2 February 2013 and 27 July 2014. The enrolling sites were the outpatient blood sampling sectors of seven hospitals located in three Italian Regions: Veneto (Negrar, San Bonifacio, and Treviso sites), Lombardia (Brescia, Mantova sites), and Friuli Venezia Giulia (Trieste, Udine sites) (Figure 1). The Centre for Tropical Diseases of Negrar (CTD) and the Health Prevention Department, Verona, were the coordinating centres.

The names of the study sites are in red.

On 1 January 2013, according to the Italian National Institute of Statistics [21], the total resident population in the six provinces of the three Regions involved in the study was 4,215,423 people (3,742,724 Italian and 472,699 foreign residents). With regard to Italian residents, 1,074,367 (28.7%) were > 60 years (born before 1952), which was the age cut off for inclusion of Italians in the present study. As for immigrants, 351,347 (74.3%) were > 17 years old, which was the age criterion for their inclusion in the present study.

### Participants

Investigators proposed the screening to individuals meeting the inclusion criteria and consecutively presenting as outpatients to perform a full blood count to one of the collaborating laboratories, during 20 randomly-selected weeks. For the study purpose, we adopted the following definitions:

Cases: individuals with peripheral eosinophil count ≥ 500/mcL;
Controls: individuals with eosinophil count < 500/mcL;
Italians: individuals born and resident in Italy;
Immigrants: individuals who were born in an endemic area and resided there for at least the first two years of life, and without Italian citizenship.

Each selected week, every centre had to recruit 10 cases and 10 controls. Inclusion criteria were: Italians born before 1952 (as in CTD experience with hundreds of patients the infection was extremely rare in younger Italian individuals with no travel history), immigrants aged ≥ 18 years. Each participant gave informed written consent. Individuals included in the study received a copy of the result of the test(s) performed and, in case of positive or uncertain result, a treatment with ivermectin (200 µg/kg, stat dose) was offered. A case report form (CRF) with essential clinical data was filled for those with positive test results.

### Laboratory methods

Screening for *S. stercoralis* infection was performed with a commercial ELISA test, IVD Research, CA, USA ELISA (IVD ELISA) during 2013, then Bordier ELISA until the end of the study period, due to unavailability of the former test; positive samples were tested with an in-house IFAT [22]. Discordant samples were analysed with Bordier ELISA during 2013, until when IVD ELISA was used as the screening test. Subsequently, due to unavailability of the latter test, Bordier ELISA was used as the screening test, and a third testing for discordant samples was no longer possible. The three tests have been described in detail elsewhere [23]. Patients testing positive in the screening tests were invited to supply a faecal sample for Koga agar plate culture for *S. stercoralis* [24] and/or for copro-parasitological test (formalin-ether concentration).

For the study purpose, patients were defined as ‘positive’ in case of two concordant positive serologic tests and/or a positive screening test AND a positive APC /copro-parasitological test. Individuals with only one positive screening test and negative stool test were classified as ‘uncertain’ in case a third serologic test was unavailable.

### Study size

There is scarce data on the prevalence of strongyloidiasis in Italy. Previous, smaller studies, found a prevalence between 10 and 15% in Italians with eosinophilia aged >60 and >68 years, respectively, and around 4% in controls of the same age group with normal eosinophil count [9,14]. On the basis of these surveys, the study size was calculated considering an odds ratio (OR) for suspected/confirmed strongyloidiasis in cases vs controls of 3, a case/control ratio of 1:1, a prevalence of strongyloidiasis in the control group of 3%, a confidence level at 95%, a study power of 80% and a design effect of 1.5. Eventually, a total of 950 Italians were to be tested, 475 cases and controls, respectively. Therefore, we initially established to enroll at least 500 individuals per group (1,000 Italian individuals in total).

The literature demonstrates a high variability in the prevalence of strongyloidiasis in immigrants, depending on their country of origin and on the screening method used [5]. Studies based on serology demonstrated a prevalence between 10 and 36%, irrespective of the eosinophil count. To calculate the study size we assumed an OR (for strongyloidiasis in cases vs controls) of 3 and a study power 80%. Based on these data, the minimum number of immigrants to be tested was 185 for each group, resulting in a total number of 370. Therefore, we attempted to enroll 200 individuals per group, 400 immigrants in total.

Overall, the minimal sample size required was of 1,400 individuals. As the sample size calculation was based on very weak estimates, particularly for Italian individuals for whom no formal, previous prevalence study was available, the proposed target sample was twice as large i.e. 2,800 individuals, 200 cases and 200 controls to be recruited by each study site.

### Statistical methods

Data at each centre were entered in a pre-structured Excel file and analysed using Stata 10 software (Stata Corp., Texas, US). For quantitative variables, data distribution was checked for normality by Shapiro-Francia test.

Since data were not normally distributed, they were analysed using the non-parametric Mann–Whitney test and the variations among groups were calculated as medians with interquartile ranges (IQR). Associations among categorical variables were analysed by Pearson’s chi-squared test or Fisher’s exact test as appropriate, and presented as observed frequencies and proportions. Trend analysis was performed by chi-squared test for linear trend. The OR of finding the outcome of interest (i.e. *S. stercoralis* infection) in relationship to the eosinophil count (defining cases and controls) and to other variables of interest (sex, age, recruitment site, geographical area of origin) were calculated by logistic regression. For all tests, the level chosen to indicate statistical significance was p < 0.05 (two-tailed).

### Ethical issues

The Ethics Committee of the coordinating centres (Comitato Etico della Provincia di Verona) approved the study protocol on 17 January 2012. The study protocol was then submitted to the Ethics Committees of each of the study sites, and formally approved.

All study participants received an information sheet and a letter for their general practitioner, explaining aim and methods of the study; signed informed consent form was required.

## Results

### Participants

**Figure 2:**
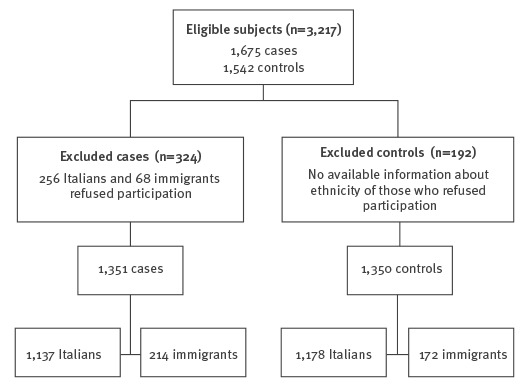
Flowchart for inclusion of participants in study of *Strongyloides stercoralis* epidemiology, northern Italy, February 2013–July 2014

A total of 3,217 individuals fulfilled the inclusion criteria; 516 were not included in the study because they were unable to give informed consent or refused to participate. The total number of individuals included in the study and analysed was 2,701 (Figure 2).

The study population comprised 1,392 men (52%) and 1,309 (48%) women. Each participating centre recruited ca 400 individuals.

Among 2,315 Italians, the proportion of women was 41% (n=464) for the 1,137 cases and 53% (n=625) for the 1,178 total controls. Median age was 73 years (range: 61–99; IQR: 67–78) and 72 years (range: 61–94; IQR: 67–77) for cases and controls, respectively. Median value of eosinophil count was 630/mcL (range: 500–24,890; IQR: 550–790) and 150/mcL (range: 0–490; IQR: 100–220) for cases and controls, respectively.

Among 386 immigrants, women represented 48% (n=103) of the 214 cases, and the proportion was higher for the 172 controls, 68% (n=117). Median age was 38 years (range: 18–87; IQR: 30–48) and 40 years (range: 18–83; IQR: 29–53) for cases and controls, respectively. Median value of eosinophil count was 655/mcL (range: 500–2,380; IQR: 570–830) and 145/mcL (range: 0–460; IQR: 70–240) for the cases and the controls, respectively.

Immigrants originated from Europe, especially eastern Europe and the Balkans (26%, n=101), Asia (22%, n=83), Sub-Saharan Africa (21%, n=82), North Africa and Middle East (18%, n=68), and Latin America (13%, n=52).

### Prevalence in cases and in controls

Overall, of 2,701 participating individuals, 149 (5%) were classified as positive (110 Italians and 39 immigrants) and 32 (1%) as uncertain (29 Italians and 3 immigrants).

Among Italians with eosinophilia (cases) 8% (97/1,137) were positive vs 1% (13/1,178) without eosinophilia. Considering a total population of the same age group of 1,074,367 in the six provinces, and an average of 4% of subjects of the same age with eosinophilia (data not shown), we obtain a rough estimate of 4,000 Italians over 60 years of age with *S. stercoralis* infection.

**Figure 3:**
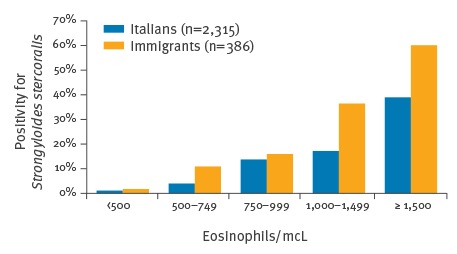
Percentage of positivity for *Strongyloides stercoralis* in relation to eosinophil count in Italians and in immigrants, study of *S. stercoralis* epidemiology, northern Italy, February 2013–July 2014 (n=2,701)

Among immigrants, positive cases were 17% (36/214) vs 2% positive controls (3/172), respectively. The proportion of positives was significantly higher among cases, both for Italians (p < 0.001) and immigrants (p < 0.001). Moreover, the higher the eosinophil count, the higher was the proportion of infected individuals, in both groups. Among Italians, the proportion of positive individuals ranged from 4% (31/780) for those with eosinophil counts between 500 and 749/mcL, to 39% (21/54) for those with eosinophil counts ≥ 1,500/mcL (p < 0.001) (Figure 3).

Among immigrants, this proportion ranged from 11% (15/138) for those with eosinophils between 500 and 749/mcL, to 60% (6/10) for those with eosinophils ≥ 1,500/mcL (p < 0.001) (Figure 3), albeit numbers were small in the latter group. Moreover, among the Italian cases, the proportion of positive individuals showed an upward trend with increasing age (p < 0.001) and varied depending on the study site (p = 0.01), with a peak in individuals born before 1936 (46/380; 12%) and in those recruited in the sites located in agricultural regions of the Po valley (e.g. San Bonifacio site: 19/146; 13%).

Immigrant cases had the following distribution, according to the region of origin of the patients: Latin America (6/31), Sub-Saharan Africa (14/48 ), Asia (11/55), Europe (2/44), and North Africa (3/36).

Some of the individuals who were positive in the screening test refused to provide a stool sample, therefore the results of stool tests were available only for 70% (104/149) of patients with positive serology of which 28% (n=29/104) had a positive stool result. Ninety-nine of 149 positive patients (66%), plus seven individuals with uncertain result, received ivermectin treatment, offered free of charge to all eligible patients. Information about possible risk factors for complicated strongyloidiasis was available for 83% (124/149) positive individuals: 16% (20/124) presented a current or past condition considered to constitute a risk for the development of severe strongyloidiasis. In the latter group, most (17/20) were treated, while two individuals refused and one died of metastatic breast cancer soon after being tested.

Analysis on the subgroup of 54 of 149 positive individuals who answered the questionnaire showed that the majority had signs and symptoms compatible with strongyloidiasis (Table 1) and had been exposed to a risk factor for infection (farm work 32/54; walking barefoot in earlier years 37/54 ). Only two of 43 responding Italians reported a stay longer than one month in endemic countries, where they might have had contact with contaminated soil. The remaining Italians did not present a relevant travel history, so we assume that the infection was probably acquired in Italy.

**Table 1:** Signs and symptoms compatible with strongyloidiasis in individuals testing positive who answered a questionnaire, study of *Strongyloides stercoralis* epidemiology, northern Italy, February 2013–July 2014 (n = 54)

Signs and symptoms	Number of Italians (%) n=43	Number of immigrants (%) n=11	Total number (%) n=54
Pruritus	23 (53.5)	4 (36.4)	27 (50)
Skin rash	13 (30.2)	2 (18.2)	15 (27.8)
Respiratory symptoms	16 (37.2)	3 (27.3)	19 (35.2)
Abdominal pain	9 (20.9)	1 (9.1)	10 (18.5)
Diarrhoea	1 (2.3)	2 (18.2)	3 (5.6)

By logistic regression, eosinophilia (p < 0.001) and immigration (p = 0.001) were independent risk factors for infection for all participants. After adjusting for birth cohort, sex and site of recruitment for Italians, or age, sex and geographical area of origin for immigrants, presence of eosinophilia ≥ 500/mcL was significantly associated with infection both in Italians (adjusted OR: 8.18; 95% CI: 4.53–14.76; p < 0.001) and in immigrants (aOR: 9.62; 95% CI: 2.85–32.41; p < 0.001). Among Italians, year of birth and site of recruitment maintained a significant association with infection also at the multivariate analysis (Table 2); the same occurred among immigrants with regard to area of origin (Table 3).

**Table 2:** Logistic regression analysis of factors associated with Italians testing positive for *Strongyloides stercoralis*, study of *S. stercoralis* epidemiology, northern Italy, February 2013–July 2014 (n = 2,315 of which 1,137 cases and 1,178 controls)

Factors	OR	95% CI	P value
Eosinophil count ≥ 500/mcL	8.18	4.53–14.76	< 0.001
Sex (male vs female)	0.93	0.63–1.39	0.730
**Year of birth**			
1947–1951	1.00	Reference	NA.
1937–1946	2.56	1.24–5.28	0.011
1936 or before	3.95	1.90–8.20	< 0.001
**Recruitment site**			
Trieste	1.00	Reference	NA
Udine	1.34	0.54–3.32	0.52
Negrar	1.67	0.69–4.00	0.25
Mantova	2.33	1.00–5.41	0.050
Brescia	2.47	1.07–5.70	0.033
Treviso	2.97	1.33–6.64	0.008
San Bonifacio	3.43	1.54–7.68	0.003

CI: confidence interval; NA: not applicable; OR: odds ratio.

**Table 3:** Logistic regression analysis of factors associated with testing positive for *Strongyloides stercoralis* among immigrant individuals, study of *S. stercoralis* epidemiology, northern Italy, February 2013–July 2014 (n = 386 of which 214 cases and 172 controls)

Factors	OR	95% CI	P value
Eosinophil count ≥ 500/mcL	9.62	2.85–32.41	< 0.001
Sex (male vs female)	1.70	0.82–3.54	0.16
Age ( + 1 year)	1.00	0.97–1.03	0.83
**Geographical area of origin**			
Europe	1.00	Reference	NA
North Africa/Middle East	1.77	0.28–11.27	0.55
Asia	5.01	1.02–24.59	0.047
Latin America	6.33	1.20–33.40	0.030
Sub-Saharan Africa	9.54	2.01–45.19	0.004

CI: confidence interval; NA: not applicable; OR: odds ratio.

## Discussion

The high number of screened individuals, especially Italians, in our study, permitted to obtain a valuable estimate of the prevalence of strongyloidiasis in the studied regions in the north of Italy: 8 and 17%, respectively, in Italians and immigrants with eosinophilia, 1 and 2% in those with a normal eosinophil count, irrespective of signs/symptoms of the infection. This finding is relevant for autochthonous Italians, for whom prevalence data were previously limited and patchy, and this study demonstrated a considerable proportion of infected individuals. In addition, 2% of Italian controls without eosinophilia with positive/uncertain test result is worth of note. The findings indicate that the infection is not an extinguished problem among elderly Italians living in the study areas.

The geographical pattern of infection prevalence is consistent with a higher transmission in agricultural areas of Po valley during the first decades of the past century, with a downward trend over time likely due to improvement of hygiene and sanitary conditions. Parts of the country, in the centre and in the south, presented in the past characteristics that make a location suitable for the free-living cycle of *S. stercoralis*. It is thus probable that a similar epidemiological picture might be prevalent in a large part, if not in the whole, of Italy. This could also be true for other countries in the Mediterranean basin, where sporadic autochthonous strongyloidiasis cases have been diagnosed [10,12].

Among immigrants, the proportion of positive individuals was high among cases with eosinophil counts ≥ 500/mcL, particularly if individuals originated from Sub-Saharan Africa, Asia, and Latin America.

Prevalence data are fundamental to implement screening and prevention programmes. We believe our results support the establishment of risk categories for screening individuals at risk of developing strongyloidiasis, such as elderly Italians (and, probably, Europeans from other Mediterranean countries) and immigrants with eosinophilia. In the latter group, it might be even cost-effective to treat all patients without testing [4**].** This should, however, be demonstrated by a well-designed study, also considering that a pre-treatment diagnostic evaluation (obligatorily including serology) is crucial to monitor cure at follow-up [25**].**

One in three of the infected individuals refused the treatment that was offered free of charge after a thorough explanation of the risk associated with untreated, chronic infection. Even general practitioners were not always keen to collaborate. Our experience suggests that strongyloidiasis is not always perceived as a relevant health problem, not only by the general population, but also by the medical community. To overcome this problem, it would be advisable to create national guidelines for the screening and management of eosinophilia that should consider strongyloidiasis among the differential diagnoses. Moreover, considering that strongyloidiasis can be fatal in immunocompromised individuals, *S. stercoralis* should be included in guidelines/protocols for screening of candidate patients for immune-suppressant therapies, such as the oncological and rheumatological ones.

### Limitations

We faced some difficulties in finding eligible immigrants for inclusion in the study, therefore the number of immigrants enrolled was slightly lower than planned. This was the reason, in addition to that provided in the Methods part, to recruit a higher number of Italians than the initially calculated sample size, as we did not deem it appropriate, to stop the recruitment in this group and continue only with immigrants. We did not include in the analysis the countries of origin as numbers for such analysis were too small and instead we analysed the continents/macro-areas. We still believe the results are useful, considering the paucity of similar data in the literature. Although the included individuals were not randomly extracted from the general population, the enrolment of out-patients, coming to the hospital laboratory to perform a very simple and common test (full blood count), results in a sample that can be comparable to the general population in that age range, in particular for the larger Italian group. The controls were unmatched, but consecutively recruited on a randomly selected day on a 1:1 basis, within the same main group (Italian or immigrant) and age range.

Finally, the accuracy of serology is high, but false-positive and false-negative results can occur [23]. The use of a second, confirmatory serological test in addition to the faecal-based tests, when available, was aimed to increase the specificity of the results. Sensitivity can be lower in immunocompromised individuals [26], however, we believe that this may have had a minimal influence on the overall results, given the high number of individuals screened [19]. The screening test had to be changed, however Bordier ELISA and IVD demonstrated similar accuracy in our previous study [23]. Therefore, we believe that the number of patients positive at screening might not have been substantially different with IVD ELISA. On the other hand, this change entailed the lack of a third serology test, therefore patients with discordant results had to be classified as uncertain. PCR was not available at our Centre before 2014, hence we could not use this method, that showed good accuracy compared with APC and Baermann technique [27,28]. PCR is less cumbersome than the traditional faecal-based methods and the samples can be stored (either frozen or with ethanol), therefore it could have been a useful tool considering the high number of individuals screened.

### Conclusions

The improvement of hygienic conditions and sanitation, and the availability of deworming drugs are likely to successfully control most helminth infections in endemic areas in the forthcoming years. However, the lack of mass drug administration programmes specifically targeting *S. stercoralis* (using ivermectin) might lead to long-term persistence of this infection in some individuals. It is also important to note that, due to the peculiarity of the auto-infective cycle of *S. stercoralis*, this parasite may remain once the other helminth infections have disappeared. This has been observed in Italy. Physicians should be aware of the categories of patients that would require screening for *S. stercoralis* infection.

## Notes

**Members of the CCM *Strongyloides* Study Group:** Fabrizio Abrescia, Alessandra Arzese, Mara Baldissera, Matteo Bassetti, Zeno Bisoffi, Dora Buonfrate, Silvio Caligaris, Giacomo Caramaschi, Francesco Castelli, Stefano Ciaffoni, Paola Coato, Mario Giobbia, Maria Gobbo, Patrizia Guzzo, Roberto Luzzati, Franco Manzato, Marta Mascarello, Cristina Maurel, Maria Merelli, Giovanna Muffato, Giuseppina Napoletano, Elena Pasqualini, Chiara Postiglione, Antonio Regazzoli, Paola Rodari, Novella Scattolo, Pier Giorgio Scotton, Anna Turrini, Gianluca Zulian.
